# Everettian relative states in the Heisenberg picture

**DOI:** 10.1098/rspa.2020.0783

**Published:** 2021-02

**Authors:** Samuel Kuypers, David Deutsch

**Affiliations:** ^1^ The Clarendon Laboratory, University of Oxford, Oxford OX1 3PU, UK; ^2^ Wolfson College, Linton Road, Oxford OX2 6UD, UK

**Keywords:** Heisenberg, quantum, local, Everett, relative states, realism

## Abstract

Everett's relative-state construction in quantum theory has never been satisfactorily expressed in the Heisenberg picture. What one might have expected to be a straightforward process was impeded by conceptual and technical problems that we solve here. The result is a construction which, unlike Everett's one in the Schrödinger picture, makes manifest the locality of Everettian multiplicity, its inherently approximative nature and its origin in certain kinds of entanglement and locally inaccessible information. (By *Everettian*, we are referring not only to Everett's own work, but also to versions of quantum theory that elaborate and refine his. The notion of relative states first appeared in Everett (Everett 1973 In *The many worlds interpretation of quantum mechanics* (eds BS DeWitt, N Graham)). We are proposing a formalism for relative states that is more detailed and more illuminating than Everett's.) Our construction also allows us to give a more precise definition of an Everett ‘universe’, under which it is fully quantum, not quasi-classical, and we compare the Everettian decomposition of a quantum state with the foliation of a space–time.

## 1. Introduction

The dynamical evolution of all quantum systems is unitary at all times except—according to most traditional ‘interpretations’ of quantum theory—during measurements. The arbitrariness, non-locality, vagueness, inelegance and anthropocentricity of this supposed exception has caused many misconceptions and confusions both in theoretical physics and in philosophy, despite the work of Everett [1], including his relative-state construction, which eliminated the exception. His primary technical innovation was to analyse quantum measurement processes by treating not only the system being measured but also the measuring apparatus or instrument (or *measurer,* for short) jointly as a unitarily evolving quantum system. As a consequence of that unitarity, when a measurer has measured an observable of another system, both the system and the measurer then exist in multiple instances such that every possible measurement outcome is observed, but in autonomously evolving parts of reality, often called ‘parallel universes’.^1^

Everett formulated his construction in the Schrödinger picture, in which the parallel universes are described by autonomously evolving components of the universal state vector. But the construction has never been satisfactorily expressed in the Heisenberg picture. This is potentially problematic for Everettian quantum theory. If, for some reason, relative states *could not* be formulated in the Heisenberg picture this might indicate that Everettian quantum theory itself is not viable. Or if a successor theory to quantum theory, such as quantum gravity, were to lack a Schrödinger picture, it would not be possible to construct an Everettian interpretation for that theory, and all those traditional problems might reappear. For instance, Dirac [2] cast doubt on the universal validity of the Schrödinger picture, arguing that ‘one can obtain a Hamiltonian which gives reasonable field equations in the Heisenberg picture, but which does not allow of solutions of the wave equation to represent physical states in the Schrödinger picture’. Furthermore, the theory of relative states itself, whether by that name or not, is central to all versions of the quantum theory of measurement, for the relative states are where the outcomes of measurements appear explicitly.

## 2. Relative states in the Schrödinger picture

In the Schrödinger picture, dynamical evolution is described by the motion of the universal state vector in Hilbert space, and the quantum-mechanical observables are fixed Hermitian operators on that space. So in the Schrödinger-picture relative-state construction, every possible outcome of the measurement of an observable appears, after a measurement, in a component of the universal state vector, and each such component consists of a product of two factors: an eigenstate of the measured observable and a state of the measurer in which there is a record of the measured eigenvalue. When these components do not subsequently undergo quantum interference, the linearity of quantum dynamics ensures that each of them evolves independently, i.e. exactly as it would if the others were not there.

For example, consider a model quantum measurement in which a measurer, initially in a Schrödinger-picture state $|M\rangle \in H_{M}$, measures the computational variable ${\hat{q}}_{z}$ of a qubit in the superposition $1/\sqrt{2}(|1+|-1\rangle )\in H_{S}$ of the two eigenstates of ${\hat{q}}_{z}$. At the initial time *t*, the composite system $H_{M}⊗H_{S}$ is in the state $|\Psi (t)\rangle =1/\sqrt{2}|M\rangle (|1\rangle +|-1\rangle )$, and the measurement, ending at time *t* + 1 and resulting in the state |Ψ(*t* + 1)〉, effects the transformation

2.1 $$\frac{1}{\sqrt{2}}|M\rangle (|1\rangle +|-1\rangle )\overset{\text{measurement}}{\to }\frac{1}{\sqrt{2}}(|M_{1}\rangle |1\rangle +|M_{-1}\rangle |-1\rangle ).$$

Here $|M_{1}\rangle$ and |*M*_−1_〉 (not necessarily distinct from or orthogonal to |*M*〉) are orthonormal eigenstates of a suitable *pointer* observable $\hat{M}$ of the measurer, whose eigenvalues *M*_1_ and *M*_−1_ are assigned the meanings ‘it was a 1’ and ‘it was a − 1’, respectively. The fact that $|M_{1}\rangle$ and $|M_{-1}\rangle$ are orthogonal, and that the final state has no components |*M*_1_〉| − 1〉 or |*M*_−1_〉| 1〉, is a token of the idealized accuracy of the measurement.

Provided there is no subsequent interference between |*M*_1_〉|1〉 and |*M*_−1_〉| − 1〉—for instance, if either the system or the measurer decoheres—the two instances of the combined system described by those two constituents will evolve independently after *t* + 1 (see, for instance, Wallace [3,4] and Zurek [5]).

The term ‘relative state’ can be used in three ways: (i) |*M*_1_〉 is the relative state of the measurer (relative to the eigenvalue 1 of the observable ${\hat{q}}_{z}$ of the qubit), and likewise for $|M_{-1}\rangle$; (ii) | 1〉 is the relative state of the qubit relative to the eigenvalue *M*_1_ of $\hat{M}$), and likewise for | − 1〉; and (iii) the product |*M*_1_|1〉 is the relative state of the combined system in which the outcome of the measurement was 1 (relative to the eigenvalues *M*_1_ and 1 of the respective observables), and likewise for *M*_−1_ and − 1.

Since the measurer, initially in state |M⟩, measures the computational variable ${\hat{q}}_{z}$, the relative states |*M*_1_〉|1〉 and |*M*_−1_〉| −1〉 of (2.1) are eigenstates of ${\hat{q}}_{z}$. These eigenstates have associated projectors

2.2 $${\hat{P}}_{1}({\hat{q}}_{z})\overset{\mathrm{def}}{=}\frac{1}{2}(\hat{1}+{\hat{q}}_{z}),\;{\hat{P}}_{-1}({\hat{q}}_{z})\overset{\mathrm{def}}{=}\frac{1}{2}(\hat{1}-{\hat{q}}_{z}),$$

where $\hat{1}$ is the unit observable of $H_{M}⊗H_{S}$. Each projector in (2.2) has an argument, namely the observable ${\hat{q}}_{z}$, and a subscript, which is an eigenvalue of ${\hat{q}}_{z}$ that specifies which eigenstate the projector projects onto.^2^ Note that the sum of projectors (2.2) equals the unit observable $\hat{1}$, so (2.2) forms a so-called projection-valued measure (PVM).

The relative states are eigenstates of ${\hat{q}}_{z}$, so we express each relative state of the model measurement (2.1) as a (normalized) component of the state vector |Ψ(t+1)⟩ in the direction specified by a projector of PVM (2.2), e.g. the relative state that contains the ‘it was a 1’ state |*M*_1_〉 is

2.3 $$\frac{{\hat{P}}_{1}({\hat{q}}_{z})|\Psi (\text{t}+1)\rangle }{|{\hat{P}}_{1}({\hat{q}}_{z})|\Psi (\text{t}+1)\rangle |}=|M_{1}\rangle |1\rangle ,$$

where the denominator ensures that (2.3) is normalized. Since the elements of a PVM sum to unity, the state vector, after a quantum measurement, can invariably be expressed as a sum of relative states, each with a complex coefficient. It is a general feature of Everett's construction that the relative states after *any* perfect measurement of a computational variable can always be defined by a PVM in this manner.^3^

The projectors (2.2) have to act on the *full* state vector in $H_{M}⊗H_{S}$ to define the relative states, and similarly, in situations where the multiverse consists not just of a measurer and a measured observable but of other systems, too, the multiverse is again decomposed into relative states through projectors acting on the full state vector. Thus, seemingly, the relative-state construction is non-local: a measurement of one system by another appears to affect the full multiverse instead of only the subsystems that participated in the measurement. Indeed, some authors—for example, Vaidman [6]—have thereby been led to conclude that an Everett universe must be spatially unbounded. In that case, Everettian quantum theory would violate Einstein's criterion of locality that ‘the real factual situation of the system *S*_2_ is independent of what is done with the system *S*_1_, which is spatially separated from the former’ (Einstein [7, p. 85]). Some Everettians—including Everett himself^4^—conclude that universes are local by the *a priori* argument that entanglement spreads through local interactions, but this does not address the point that the Schrödinger-picture relative states are not the products of local states and so apparently violate Einstein's criterion. In other words, Schrödinger-picture relative states are non-local because the Schrödinger picture is not a local formalism [8]. Such non-local *formalisms* are generically admitted by both local and non-local *theories* of the world. Quantum theory describes a local world because it also admits a local formalism: as we shall show in §4, in the Heisenberg picture, our relative-state construction is manifestly local and, therefore, ‘universes’ are always spatially bounded.

Formulating the relative-state construction within the Heisenberg picture is important because, for instance, information flow in quantum systems can only be analysed transparently in the Heisenberg picture [9,10]. In the Heisenberg picture, information has a well-defined location in physical systems [8,11]. Related ideas have been explored in the Heisenberg picture—for example, Wallace [4] has used the Heisenberg picture to give an account of decoherent histories, but he did not specify what the Heisenberg-picture relative states are in that picture. A construction similar to ours was presented by Hewitt-Horsman & Vedral [12], in their Section V. But, they do not draw the conclusion that the Heisenberg relative states are states of fully quantum systems, with their own algebras of observables and with the identical structure to those of the original system; nor do they explain that these systems are always local in space and are, therefore, not literally universes.

## 3. Quantum computation in the Heisenberg picture

In contrast with the formalism of the Schrödinger picture, that of the Heisenberg picture consists of *evolving* q-number observables and a *fixed* state vector (or in the case of subsystems, a fixed density operator). Because of this difference, certain important properties of quantum systems are transparent in the Heisenberg picture but opaque in the Schrödinger picture. In particular, in the Heisenberg picture, the time-varying entities, the Heisenberg observables, are local, and affect each other only locally, just as in classical physics, unlike the time-varying state vector of the Schrödinger picture, which is non-local. This implies that, in the Heisenberg picture, all physical information is assigned a location, even in entangled systems; see Deutsch & Hayden [9].

We shall construct the Heisenberg relative states for a quantum computational network; this technique, as used by Deutsch & Hayden [9], ensures that our results about relative states generalize to all quantum systems. This is not because quantum computational networks are the most general system—a network consists exclusively of qubits—but since such networks are universal in their capacity to simulate with arbitrary accuracy any other physical system, any result obtained about relative states in these networks must be a general feature of quantum theory.^5^

Accordingly, consider a quantum computational network N consisting of *n* interacting qubits $Q_{1}$, $Q_{2},\ldots ,Q_{n}$. Following Gottesman [13] and Deutsch & Hayden [9], we represent each qubit at the initial time *t* as a triple of its observables, which we call the *descriptors* of the qubit

3.1 $${\hat{q}}_{a}^{\left(t\right)}=({\hat{q}}_{ax}^{\left(t\right)},\;{\hat{q}}_{ay}^{\left(t\right)},\;{\hat{q}}_{az}^{\left(t\right)}).$$

They satisfy the algebraic relations

3.2 $$\begin{array}{ll} {\hat{q}}_{ai}^{\left(t\right)}{\hat{q}}_{aj}^{\left(t\right)}=\delta_{ij}\hat{1}+i{\epsilon_{ij}}^{k}{\hat{q}}_{ak}^{\left(t\right)} & (i,j,k\in \{x,y,z\}),\\ {}[{\hat{q}}_{a}^{\left(t\right)},{\hat{q}}_{b}^{\left(t\right)}]=0 & (a\neq b). \end{array}\}$$

When an index appears twice in a product, once as a superscript and once as a subscript, as *k* does in (3.2), we implicitly sum it over all its possible values, in accordance with the Einstein summation convention. Here, the values summed over are {*x*, *y*, *z*}.

From (3.2), it follows that, for a network of *n* qubits, each qubit descriptor can be expressed as a Hermitian 2^*n*^ × 2^*n*^ matrix, so, for instance, the descriptors of a network consisting of a single qubit can be taken to have the initial matrix representation

3.3 $${\hat{q}}_{1x}^{\left(0\right)}={\hat{\sigma }}_{x}\overset{\mathrm{def}}{=}\left(\begin{array}{cc} 0 & 1\\ 1 & 0 \end{array}\right);\;{\hat{q}}_{1y}^{\left(0\right)}={\hat{\sigma }}_{y}\overset{\mathrm{def}}{=}\left(\begin{array}{cc} 0 & -i\\ i & 0 \end{array}\right);\;{\hat{q}}_{1z}^{\left(0\right)}={\hat{\sigma }}_{z}\overset{\mathrm{def}}{=}\left(\begin{array}{cc} 1 & 0\\ 0 & -1 \end{array}\right).$$

For the *n*-qubit network N, the initial representation (3.3) generalizes to

3.4 $${\hat{q}}_{a}^{\left(0\right)}={\hat{1}}^{\text{a}-1}⊗\hat{\sigma }⊗{\hat{1}}^{n-a},$$

where ${\hat{1}}^{k}$ is a tensor product of *k* single-qubit identity operators, and $\hat{\sigma }=({\hat{\sigma }}_{x},{\hat{\sigma }}_{y},{\hat{\sigma }}_{z})$. The initial matrix representation (3.4) of the descriptors will be altered by the subsequent evolution of the network, but since the descriptors of N evolve unitarily the algebra (3.2) is a constant of the motion.

It is convenient and conventional, and loses no generality, to assume that each gate in a network requires exactly 1 unit of time to act, so that the computational state of the network need only be specified at integer values of time. The effect of a general quantum gate **G** on $Q_{a}$ after one such period is

3.5 $${\hat{q}}_{a}^{\left(t+1\right)}=U_{\text{G}}^{†}({\hat{q}}_{1}^{\left(t\right)},\ldots ,{\hat{q}}_{n}^{\left(t\right)})\;{\hat{q}}_{a}^{\left(t\right)}U_{\text{G}}({\hat{q}}_{1}^{\left(t\right)},\ldots ,{\hat{q}}_{n}^{\left(t\right)}).$$

Here, the unitary *U*_**G**_ implements the gate **G** and is a function of the time-dependent descriptors of N. Note that, in the Heisenberg picture, *U*_**G**_ generally varies with time and, therefore, its matrix representation in our basis changes with time as well, but it is a fixed (i.e. characteristic of the gate) function of the time-dependent descriptors. For example, the **not** gate is a single-qubit gate which, when it acts on qubit $Q_{a}$, toggles the value of its *z*-observable when it is sharp; the definition of the **not** gate in terms of the descriptors is

3.6 $$U_{\text{not},\;a}({\hat{q}}_{1}^{\left(t\right)},\ldots ,{\hat{q}}_{n}^{\left(t\right)})={\hat{q}}_{ax}^{\left(t\right)}.$$

The only gate whose matrix representation in the Heisenberg picture is time-independent is the ‘unit wire’ **I**, the effect of which is that it leaves the descriptors of N unchanged, and for a network of *n* qubits has matrix representation $U_{\text{I}}={\hat{1}}^{n}$.

Since the unitary of a gate can be expressed as a characteristic *function* of the descriptors, a gate also has a characteristic *effect* on those descriptors, which can be specified algebraically. This provides a complementary way of defining a gate in the Heisenberg picture. For example, using (3.5) and (3.6), we find that, after the application of the **not** gate, the descriptors of $Q_{a}$ at time *t* + 1 have the following fixed expression in terms of its descriptors at time *t*:

3.7 $$\text{not}:\;{\hat{q}}_{a}^{\left(t+1\right)}\;=({\hat{q}}_{ax}^{\left(t\right)},\;-{\hat{q}}_{ay}^{\left(t\right)},\;-{\hat{q}}_{az}^{\left(t\right)}),$$

while all other qubits in the network are unaffected. Expressing gates in form (3.7) rather than (3.6) often makes their effect on a network more transparent. For example, it is manifest in (3.7) that the eigenvalues of the *z*-observable of $Q_{a}$ are toggled by the **not** gate, as ${\hat{q}}_{az}^{\left(t+1\right)}=-{\hat{q}}_{az}^{\left(t\right)}$.

N is fully specified by its time-dependent descriptors and constant Heisenberg state |Ψ⟩. We shall fix the Heisenberg state to be a standard constant by using the basis $\left\{|{\hat{q}}_{1z},\ldots ,{\hat{q}}_{nz};t\rangle \right\}$ of instantaneous eigenstates of the descriptors $\left\{{\hat{q}}_{az}^{\left(t\right)}\right\}$ so that |Ψ〉 equals |1, … ,1;*t* = 0〉. Then the expectation value of an arbitrary time-dependent observable $\hat{A}(t)$ of N is

3.8 $$\langle \hat{A}(t)\rangle \overset{\mathrm{def}}{=}\langle \Psi |\hat{A}(t)|\Psi \rangle \overset{\mathrm{def}}{=}\langle 1,\ldots ,1;0|\hat{A}(t)|1,\ldots ,1;0\rangle .$$

Since the Heisenberg ‘state’ |Ψ〉 is a fixed constant, it contains no information about the physical state of N. All such information resides instead in the Heisenberg observables of N.

Whenever the expectation value of an observable $\hat{A}(t)$ of N has the property that $\left\langle \hat{A}{\left(t\right)}^{2}=\hat{A}{\left(t\right)}^{2}\right\rangle$, the Heisenberg state is an eigenstate of $\hat{A}(t)$ and the observable is then said to be *sharp* at time *t* with the value $\left\langle \hat{A}(t)\right\rangle$ (in Everettian terms, it has ‘the same value in all universes' at time *t*), where that value is one of the eigenvalues of $\hat{A}(t)$.

In the Heisenberg picture, two qubits $Q_{a}$ and $Q_{b}$ are entangled at time *t* if there exists a pair of descriptors ${\hat{q}}_{ai}^{\left(t\right)}$ and ${\hat{q}}_{bj}^{\left(t\right)}$ such that $\left\langle {\hat{q}}_{ai}^{\left(t\right)}{\hat{q}}_{bj}^{\left(t\right)}\right\rangle \neq \left\langle {\hat{q}}_{ai}^{\left(t\right)}\right\rangle \left\langle {\hat{q}}_{bj}^{\left(t\right)}\right\rangle$ [14]. An entangled quantum system is capable of storing *locally inaccessible information*—information that cannot be recovered through any measurement of that quantum system alone [9]. This fact is transparent in the Heisenberg picture, since it makes the location of all physical information explicit. For instance, consider two entangled qubits $Q_{a}$ and $Q_{b}$ that, at the initial time *t*, have expectation values $\left\langle {\hat{q}}_{a}^{\left(t\right)}\right\rangle =(0,0,0)$ and $\left\langle {\hat{q}}_{b}^{\left(t\right)}\right\rangle =(0,0,0)$ and $\left\langle {\hat{q}}_{az}^{\left(t\right)}{\hat{q}}_{bz}^{\left(t\right)}\right\rangle =1$, so the qubits are in a Bell state. Then, at time *t*, $Q_{a}$ effects a gate *R_z_*(*θ*), which rotates $Q_{a}$ by an angle of *θ* radians about its *z*-axis. That is to say, the descriptors of $Q_{a}$ evolve as follows:

3.9 $${\hat{q}}_{a}^{\left(t+1\right)}\;=(\cos\theta {\hat{q}}_{ax}^{\left(t\right)}-\sin\theta {\hat{q}}_{ay}^{\left(t\right)},\;\cos\theta {\hat{q}}_{ay}^{\left(t\right)}+\sin\theta {\hat{q}}_{ax}^{\left(t\right)},\;{\hat{q}}_{az}^{\left(t\right)}).$$

Manifestly, the information about the angle *θ* is located in the *x*- and *y*-observables of ${\hat{q}}_{a}^{\left(t+1\right)}$, yet none of the expectation values of the qubit's observables reflect the fact that it contains this information since

3.10 $$\left\langle {\hat{q}}_{a}^{\left(t+1\right)}\right\rangle =(0,0,0).$$

Thus, the angle *θ* is *locally inaccessible*—i.e. the results of measurements made on $Q_{a}$ alone, by measurers with no prior knowledge of *θ*, reveal nothing about *θ*, even statistically (if the whole experiment preparing $Q_{a}$ is repeated any number of times). As we shall show in §5, owing to entanglement, instances of a system in a relative state are also capable of storing locally inaccessible information.

## 4. The Heisenberg-picture relative-state construction

When the relative-state construction is formulated in the Schrödinger picture, the joint state vector of the observed system and the measurer are decomposed into *relative-state vectors*, each describing a ‘universe’.^6^ Although translating between the Schrödinger and Heisenberg pictures is usually a straightforward process, doing so for the relative-state construction brings with it the conceptual difficulty of decomposing the Heisenberg descriptors into *relative descriptors*: q-numbers which, following a measurement, represent individual instances of a combined quantum system, and which should correspond to the Schrödinger-picture relative-state vectors. Our construction in what follows resolves that difficulty.

Consider a measurement performed by a network M that consists of the two qubits $Q_{M}$ and $Q_{S}$, where $Q_{M}$ represents an idealized measurer that is programmed to measure the *z*-observable of system $Q_{S}$. The qubits in M have initial (*t* = 0) matrix representation (3.4) with *n* = 2

4.1 $$\begin{array}{rl} {\hat{q}}_{S}^{\left(0\right)} & =({\hat{\sigma }}_{x}⊗\hat{1},{\hat{\sigma }}_{y}⊗\hat{1},{\hat{\sigma }}_{z}⊗\hat{1}),\\ {\hat{q}}_{M}^{\left(0\right)} & =(\hat{1}⊗{\hat{\sigma }}_{x},\hat{1}⊗{\hat{\sigma }}_{y},\hat{1}⊗{\hat{\sigma }}_{z}). \end{array}\}$$

To reduce notational clutter, we shall abbreviate this as

4.2 $$\begin{array}{rl} {\hat{q}}_{S} & =({\hat{q}}_{Sx},{\hat{q}}_{Sy},{\hat{q}}_{Sz}),\\ {\hat{q}}_{M} & =({\hat{q}}_{Mx},{\hat{q}}_{My},{\hat{q}}_{Mz}). \end{array}\}$$

The Heisenberg state |Ψ〉 of the network equals |1, 1;0〉, so that, at *t* = 0, the *z*-observables of both $Q_{M}$ and $Q_{S}$ are sharp with value 1. The relative-state construction explains why a unitarily evolving measurer appears to observe only one of the eigenvalues of an observable that is non-sharp: the other eigenvalues occurred in different ‘universes’. To elaborate this, we apply a Hadamard gate **H** to $Q_{S}$ before the measurement begins. **H** is a single-qubit gate which rotates a qubit by *π* radians about the axis that bisects the angle between the *x*- and *z*-axis. That is to say, when **H** is applied to $Q_{S}$, it has the effect

4.3 $$\begin{array}{rl} {\hat{q}}_{S}^{\left(1\right)} & =({\hat{q}}_{Sz},-{\hat{q}}_{Sy},{\hat{q}}_{Sx}),\\ {\hat{q}}_{M}^{\left(1\right)} & =({\hat{q}}_{Mx},{\hat{q}}_{My},{\hat{q}}_{Mz}). \end{array}\}$$

Thus, at *t* = 1, the Heisenberg state is not an eigenstate of ${\hat{q}}_{Sz}^{\left(1\right)}$, so this descriptor has no sharp value.

Next, between *t* = 1 and *t* = 2, $Q_{M}$ performs an idealized measurement of the *z*-observable of $Q_{S}$. This is implemented by the two-qubit ‘controlled-not’ (or ‘perfect measurement’) gate **cnot**. The qubits that this gate acts on are known as the *control* and *target* qubits, which in this case are $Q_{S}$ and $Q_{M}$, respectively. The effect of a **cnot** gate depends on the value of the control qubit: at a general time *t*, **cnot** has no effect on the target if ${\hat{q}}_{\text{S}z}^{\left(t\right)}$ of the control is sharp with value 1 and performs a **not** operation on the target if ${\hat{q}}_{\text{S}z}^{\left(t\right)}$ of the control is sharp with value − 1. It follows that the unitary *U*_**cnot**_ that implements the **cnot** operation at a general time *t* is

4.4 $$U_{\text{cnot}}({\hat{q}}_{S}^{\left(t\right)},{\hat{q}}_{M}^{\left(t\right)})={\hat{P}}_{1}({\hat{q}}_{Sz}^{\left(t\right)})+U_{\text{not},M}({\hat{q}}_{M}^{\left(t\right)}){\hat{P}}_{-1}({\hat{q}}_{Sz}^{\left(t\right)}).$$

The operators ${\hat{P}}_{1}({\hat{q}}_{Sz}^{\left(t\right)})=\frac{1}{2}(\hat{1}+{\hat{q}}_{Sz}^{\left(t\right)})$ and ${\hat{P}}_{-1}({\hat{q}}_{Sz}^{\left(t\right)})=\frac{1}{2}(\hat{1}-{\hat{q}}_{Sz}^{\left(t\right)})$ are the Heisenberg equivalent of the projectors of (2.2), and their appearance in (4.4) makes explicit that **cnot** is a conditional **not** operation.

By using (3.5) and (4.4), we find that the effect of the **cnot** gate on the descriptors of the two qubits at a general time *t* is

4.5 $$\left\{\begin{array}{c} {\hat{q}}_{S}^{\left(t+1\right)}\\ {\hat{q}}_{M}^{\left(t+1\right)} \end{array}\right\}=\left\{\begin{array}{c} \left({\hat{q}}_{Sx}^{\left(t\right)}{\hat{q}}_{Mx}^{\left(t\right)},\;{\hat{q}}_{Sy}^{\left(t\right)}{\hat{q}}_{Mx}^{\left(t\right)},\;{\hat{q}}_{Sz}^{\left(t\right)}\right)\\ \left({\hat{q}}_{Mx}^{\left(t\right)},\;{\hat{q}}_{My}^{\left(t\right)}{\hat{q}}_{Sz}^{\left(t\right)},\;{\hat{q}}_{Mz}^{\left(t\right)}{\hat{q}}_{Sz}^{\left(t\right)}\right) \end{array}\right\}.$$

If both descriptors ${\hat{q}}_{Sz}^{\left(t\right)}$ and ${\hat{q}}_{Mz}^{\left(t\right)}$ of (4.5) are initially sharp and, in particular, if $\left\langle {\hat{q}}_{Mz}^{\left(t\right)}\right\rangle =1$, then the **cnot** evolves the value of ${\hat{q}}_{Mz}^{\left(t\right)}$ into a ‘copy’ of the value of ${\hat{q}}_{Sz}^{\left(t\right)}$—that is to say, $\left\langle {\hat{q}}_{Mz}^{\left(t+1\right)}\right\rangle =\left\langle {\hat{q}}_{Sz}^{\left(t\right)}\right\rangle =\pm 1$. Accordingly, after the application of **cnot**, the sharp values 1 and − 1 of ${\hat{q}}_{Mz}^{\left(t\right)}$ have the unequivocal physical meaning ‘the control was a 1’ and ‘the control was a − 1’. If ${\hat{q}}_{Sz}^{\left(t\right)}$ is non-sharp, and ${\hat{q}}_{Mz}^{\left(t\right)}$ is sharp with value 1, then a **cnot** operation still ‘copies’ the value of a superposed control onto ${\hat{q}}_{Mz}^{\left(t\right)}$ by evolving the target into a mixed state, and the combined system is then entangled.

Note in passing, by inspection of (4.5), that the **cnot** gate *also* effects a perfect measurement of the *x*-observable of $Q_{M}$, storing the result in the *x*-observable of $Q_{S}$; in that interpretation of what the gate does, the roles of ‘target’ and ‘control’ are interchanged. In general, whether a system is being measured or performs a measurement depends not only on the interaction between them but also on how the information in each system is *subsequently* used, and, in general, it cannot be freely chosen which information stored in the pair of qubits to use. Decoherence would, for example, determine which of the qubits in (4.5) can act as a measurer [5,17].

Using (4.3) and (4.5), we find that the descriptors at *t* = 2, after the application of the **cnot** gate, are

4.6 $$\begin{array}{rl} {\hat{q}}_{S}^{\left(2\right)} & =({\hat{q}}_{Sz}{\hat{q}}_{Mx},-{\hat{q}}_{Sy}{\hat{q}}_{Mx},{\hat{q}}_{Sx}),\\ {\hat{q}}_{M}^{\left(2\right)} & =({\hat{q}}_{Mx},{\hat{q}}_{My}{\hat{q}}_{Sx},{\hat{q}}_{Mz}{\hat{q}}_{Sx}). \end{array}\}$$

The Schrödinger state vector of the qubits at *t* = 2 is

4.7 $$|\Psi (2)=\frac{1}{\sqrt{2}}(|1\rangle |1+|-1|-1\rangle ),$$

with relative states |1〉|1〉 and | − 1〉| − 1〉. To construct the corresponding relative-state decomposition *of the descriptors* (4.6), we use (4.4) to express the descriptors of $Q_{M}$ at *t* = 2 in terms of descriptors at *t* = 1

4.8 $$\begin{array}{rl} {\hat{q}}_{M}^{\left(2\right)} & =U_{\text{cnot}}^{†}({\hat{q}}_{S}^{\left(1\right)},{\hat{q}}_{M}^{\left(1\right)}){\hat{q}}_{M}^{\left(1\right)}U_{\text{cnot}}({\hat{q}}_{S}^{\left(1\right)},{\hat{q}}_{M}^{\left(1\right)})\\ & ={\hat{q}}_{M}^{\left(1\right)}{\hat{P}}_{1}({\hat{q}}_{Sz}^{\left(1\right)})+[U_{\text{not, }M}^{†}({\hat{q}}_{M}^{\left(1\right)}){\hat{q}}_{M}^{\left(1\right)}U_{\text{not, }M}({\hat{q}}_{M}^{\left(1\right)})]{\hat{P}}_{-1}({\hat{q}}_{\text{S}z}^{\left(1\right)}). \end{array}$$

Equation (4.8) shows the **cnot** ‘splitting’ $Q_{M}$ into two simultaneously existing *instances*: one of them participates in a **not** operation, while the other is left unchanged. Let us call these two instances $Q_{M,1}$ and $Q_{M,-1}$, where the additional subscript specifies whether the instance is relative to the measured value 1 or − 1. The instances $Q_{M,1}$ and $Q_{M,-1}$ are in the respective Heisenberg-picture relative states and are defined as

4.9 $$\begin{array}{rl} Q_{M,1} & :\;{\hat{q}}_{M,1}^{\left(2\right)}\overset{\mathrm{def}}{=}{\hat{q}}_{M}^{\left(2\right)}{\hat{P}}_{1}({\hat{q}}_{Sz}^{\left(2\right)}),\\ Q_{M,-1} & :{\hat{q}}_{M,-1}^{\left(2\right)}\overset{\mathrm{def}}{=}{\hat{q}}_{M}^{\left(2\right)}{\hat{P}}_{-1}({\hat{q}}_{Sz}^{\left(2\right)}). \end{array}\}$$

Because of relations (3.2), the projectors ${\hat{P}}_{1}({\hat{q}}_{Sz}^{\left(2\right)})$ and ${\hat{P}}_{-1}({\hat{q}}_{Sz}^{\left(2\right)})$ commute with ${\hat{q}}_{M}^{\left(2\right)}$. This ensures that the order of the factors in (4.9) makes no difference and that ${\hat{q}}_{M,1}^{\left(2\right)}$ and ${\hat{q}}_{M,-1}^{\left(2\right)}$ are Hermitian. Also, ${\hat{q}}_{Sz}^{\left(1\right)}={\hat{q}}_{Sz}^{\left(2\right)}$, and so there is no discrepancy between the definitions of ${\hat{q}}_{M,1}^{\left(2\right)}$ and ${\hat{q}}_{M,-1}^{\left(2\right)}$ in (4.8) and (4.9).

$Q_{M,1}$ and $Q_{M,-1}$ each hold a record of a different eigenvalue of ${\hat{q}}_{\text{S}z}$, which is why the instances $Q_{M,1}$ and $Q_{M,-1}$ are in distinct *histories* of the measurer, just as in the Schrödinger-picture relative state. Hence, we shall call the descriptors of $Q_{M,1}$ and $Q_{M,-1}$ the *relative descriptors* (or descriptors relative to the results 1 and − 1, etc.).

The relative descriptors satisfy an appropriate form of the Pauli algebra (3.3). For example, note that the squares of the relative descriptors of $Q_{M,1}$ do not equal the unit observable $\hat{1}$ of the full algebra of operators since ${\left({\hat{q}}_{(M,1)i}\right)}^{2}={\hat{P}}_{1}({\hat{q}}_{\text{S}z})$; moreover, ${\hat{P}}_{1}({\hat{q}}_{\text{S}z})\;{\hat{q}}_{(M,1)i}={\hat{q}}_{(M,1)i}{\hat{P}}_{1}({\hat{q}}_{\text{S}z})\;={\hat{q}}_{(M,1)i}$ for all *i* ∈ {*x*, *y*, *z*}. (Here, we have dropped the time labels for clarity.) Because of these algebraic relations, ${\hat{1}}_{M,1}\overset{\mathrm{def}}{=}{\hat{P}}_{1}({\hat{q}}_{\text{S}z})$ is the unit observable in the relative algebra of $Q_{M,1}$, and, similarly, ${\hat{1}}_{M,-1}\overset{\mathrm{def}}{=}{\hat{P}}_{-1}({\hat{q}}_{\text{S}z})$ is the unit observable in the relative algebra of $Q_{M,-1}$. Physically, this means that relative descriptors adhere to the Pauli algebra, and $Q_{M,1}$ and $Q_{M,-1}$ are, thus, fully fledged qubits

4.10 $$\begin{array}{rl} {\hat{q}}_{(M,1)i}{\hat{q}}_{(M,1)j} & =\delta_{ij}{\hat{1}}_{M,1}+i{\epsilon_{ij}}^{k}{\hat{q}}_{(M,1)k},\\ {\hat{q}}_{(M,-1)i}{\hat{q}}_{(M,-1)j} & =\delta_{ij}{\hat{1}}_{M,-1}+i{\epsilon_{ij}}^{k}{\hat{q}}_{(M,-1)k}, \end{array}\}\;(i,j,k\in \{x,y,z\}).$$

So, in each Everettian ‘universe’, quantum theory holds in full, but the matrix representation required to represent separately the sets of relative descriptors is of a smaller dimension than that of the full descriptors of $Q_{M}$.

Because $Q_{S}$ also contains a copy of ${\hat{q}}_{Mz}^{\left(2\right)}$, there is a similar relative-state description of $Q_{S}$ relative to the values 1 and − 1 of ${\hat{q}}_{Mz}^{\left(2\right)}$. These instances of $Q_{S}$ are

4.11 $$\begin{array}{rl} Q_{S,1} & :{\hat{q}}_{S,1}^{\left(2\right)}\overset{\mathrm{def}}{=}{\hat{q}}_{S}^{\left(2\right)}{\hat{P}}_{1}({\hat{q}}_{Mz}^{\left(2\right)}),\\ Q_{S,-1} & :{\hat{q}}_{S,-1}^{\left(2\right)}\overset{\mathrm{def}}{=}{\hat{q}}_{S}^{\left(2\right)}{\hat{P}}_{-1}({\hat{q}}_{Mz}^{\left(2\right)}). \end{array}\}$$

The order of the factors in (4.11) is again irrelevant since the observables of $Q_{M}$ and $Q_{S}$ commute. And the instances $Q_{S,1}$ and $Q_{S,-1}$ again obey the appropriate form of the Pauli algebra, but now with relative unit observables ${\hat{1}}_{S,1}\overset{\mathrm{def}}{=}{\hat{P}}_{1}({\hat{q}}_{Mz}^{\left(2\right)})$ and ${\hat{1}}_{S,-1}\overset{\mathrm{def}}{=}{\hat{P}}_{-1}({\hat{q}}_{Mz}^{\left(2\right)})$.

Analogously to the Schrödinger-picture relative-state construction, the projectors ${\hat{P}}_{1}({\hat{q}}_{Sz}^{\left(2\right)})$ and ${\hat{P}}_{-1}({\hat{q}}_{Sz}^{\left(2\right)})$ of the measured observable form a PVM, and the relative descriptors (4.9) of $Q_{M}$ are defined as products of ${\hat{q}}_{M}^{\left(2\right)}$ and the projectors of this PVM. Since quantum computational networks are universal, it must be a general feature of the Heisenberg relative-state construction that the relative descriptors of *any* measurer, after a perfect measurement, are the absolute descriptors projected via a PVM in the above manner. This resolves the conceptual difficulty of expressing the Heisenberg descriptors in terms of relative descriptors, and, importantly, our solution is also in agreement with Hewitt-Horsman & Vedral [12].

Borrowing a term from differential geometry, we say that entangled systems, such as $Q_{M}$ and $Q_{S}$, can be *foliated* into relative states. In the Heisenberg picture, such a foliation is always spatially *local*^7^ since only those systems that are entangled as a result of a measurement are foliated into relative states, whereas no other systems are affected. This is evident from the fact that the foliations of $Q_{M}$ and $Q_{S}$ are individually specified, the latter in (4.11) and the former in (4.9). By contrast, the Schrödinger picture, which is not a local description of quantum systems, does not allow systems to be foliated individually: a composite system can only be foliated jointly by a projector operating on the full Schrödinger state vector. So, in the Heisenberg picture, unentangled systems cannot be foliated into physically meaningful relative states.

Foliating a quantum system changes the expectation value in relative states from those in the absolute network M. For example, at time *t* = 2, the conditional expectation value for an arbitrary descriptor ${\hat{q}}_{Mi}^{\left(2\right)}$ of $Q_{M}$ given that the *z*-observable of $Q_{S}$ was measured to be a 1 is

4.12 $${\left\langle \hat{q}{\;}_{Mi}^{\left(2\right)}\right\rangle }_{M,1}\overset{\mathrm{def}}{=}\frac{\left\langle {\hat{q}}_{Mi}^{\left(2\right)}{\hat{P}}_{1}({\hat{q}}_{Sz}^{\left(2\right)})\right\rangle }{\left\langle {\hat{P}}_{1}({\hat{q}}_{Sz}^{\left(2\right)})\right\rangle }.$$

Thus, so long as no further entangling or unentangling interactions take place, the expectation values of $Q_{M,1}$ (and any other system in a relative state, relative to the result 1 of our measurement) are the same as they would be *if* the Heisenberg state had changed during the measurement,

4.13 $$|\Psi \rangle \to |\Psi_{M,1}\rangle \overset{\mathrm{def}}{=}\frac{{\hat{P}}_{1}({\hat{q}}_{Sz}^{\left(2\right)})\text{|}\Psi \rangle }{|{\hat{P}}_{1}({\hat{q}}_{Sz}^{\left(2\right)})\text{|}\Psi \rangle \text{|}}\text{.}$$

We call |*Ψ_M_*_,1_〉 the *relative Heisenberg state*, relative to the result 1 of the measurement of ${\hat{q}}_{Sz}^{\left(2\right)}$. Note that the normalization factor $\left\langle {\hat{P}}_{1}({\hat{q}}_{Sz}^{\left(2\right)})\right\rangle$ is necessarily non-zero, as ${\hat{q}}_{Sz}^{\left(2\right)}$ is non-sharp.

Since the expectation values of the relative descriptors differ from those of the observables of the absolute (multiversal) network M, it is possible for an observable, such as ${\hat{q}}_{Mz}^{\left(2\right)}$, not to be sharp with respect to the absolute network (i.e. $\left\langle {\hat{q}}_{Mz}^{\left(2\right)}\right\rangle =0$) but for ${\hat{q}}_{(M,1)z}^{\left(2\right)}$ to be sharp *relative* to ${\hat{q}}_{Sz}^{\left(2\right)}$ having been measured with value 1 (i.e. ${\left\langle {\hat{q}}_{(M,1)z}^{\left(2\right)}\right\rangle }_{M,1}=1$). We can describe this informally as ${\hat{q}}_{Mz}^{\left(2\right)}$ having a sharp value in each universe (or in each relative state) but not in the multiverse at large.

## 5. Everettian ‘universes’

The relative-state construction, in any picture, is a calculus for expressing a quantum system in terms of the behaviour of its constituent instances in situations where some or all of those instances evolve autonomously. In such situations, the system consists of parallel ‘histories’ or ‘universes’, each represented by a relative state. However, in generic situations, a foliation into such universes does not exist. Whether it does depends on the dynamical evolution of the entangled system.

To study the types of dynamical evolution that permit an entangled system to be foliated into parallel ‘universes’, let us once more consider the quantum computational network M at *t* = 2, when the network is foliated into $Q_{S,1}$ with $Q_{M,1}$, and $Q_{S,-1}$ with $Q_{M,-1}$. This foliation is possible because, immediately after the measurement, $Q_{M}$ contains a *copy* of the descriptor ${\hat{q}}_{Sz}^{\left(2\right)}$.^8^ We say that a qubit like $Q_{M}$ contains a copy of a descriptor of another qubit, such as ${\hat{q}}_{Sz}^{\left(2\right)}$, if the product of an observable of $Q_{M}$ and ${\hat{q}}_{Sz}^{\left(2\right)}$ is sharp (e.g. ${\hat{q}}_{Mz}^{\left(2\right)}{\hat{q}}_{Sz}^{\left(2\right)}$ is sharp with value 1) and all descriptors of $Q_{M}$ commute with ${\hat{q}}_{Sz}^{\left(2\right)}$ (this second condition ensures that ${\hat{q}}_{Sz}^{\left(2\right)}$ is not one of the descriptors of $Q_{M}$, in which case it would trivially contain a copy of that descriptor). If at a more general time *t* ≥ 2, $Q_{M}$ still contains a copy of ${\hat{q}}_{Sz}^{\left(2\right)}$, the qubit decomposes into the instances

5.1 $$\begin{array}{rl} Q_{M,1} & :{\hat{q}}_{M,1}^{\left(t\right)}\overset{\mathrm{def}}{=}{\hat{q}}_{M}^{\left(t\right)}{\hat{P}}_{1}({\hat{q}}_{Sz}^{\left(2\right)}),\\ Q_{M,-1} & :{\hat{q}}_{M,-1}^{\left(t\right)}\overset{\mathrm{def}}{=}{\hat{q}}_{M}^{\left(t\right)}{\hat{P}}_{-1}({\hat{q}}_{Sz}^{\left(2\right)}). \end{array}\}$$

Likewise, by applying our definition to $Q_{S}$, we find that, at *t* = 2, it contains a copy of ${\hat{q}}_{Mz}^{\left(2\right)}$, and if $Q_{S}$ retains this copy at a more general time *t* ≥ 2, then the qubit can be foliated into the relative states

5.2 $$\begin{array}{rl} Q_{S,1} & :{\hat{q}}_{S,1}^{\left(t\right)}\overset{\mathrm{def}}{=}{\hat{q}}_{S}^{\left(t\right)}{\hat{P}}_{1}({\hat{q}}_{Mz}^{\left(2\right)}),\\ Q_{S,-1} & :{\hat{q}}_{S,-1}^{\left(t\right)}\overset{\mathrm{def}}{=}{\hat{q}}_{S}^{\left(t\right)}{\hat{P}}_{-1}({\hat{q}}_{Mz}^{\left(2\right)}). \end{array}\}$$

The gates that cause these relative states of M to evolve autonomously will, owing to the universality of quantum computational networks, also model when a general quantum system can be foliated into ‘universes’. A qubit $Q_{a}$ evolves autonomously between *t* and *t* + 1 if its descriptors ${\hat{q}}_{a}^{\left(t+1\right)}$ depend solely on ${\hat{q}}_{a}^{\left(t\right)}$, and similarly a relative state evolves autonomously if its relative descriptors at time *t* + 1 depend only on those same relative descriptors at time *t*. Using this definition, which class of gates, acting between *t* = 2 and *t* = 3, would ensure that the relative states of M evolve autonomously between those times? Evidently, not all gates do since, for instance, a second application of **cnot** would erase the information about $Q_{S}$ contained in $Q_{M}$, so this gate would destroy the relative-state structure. On the other hand, any gate that does not affect ${\hat{q}}_{Sz}^{\left(2\right)}$ and ${\hat{q}}_{Mz}^{\left(2\right)}$ will ensure that the relative states evolve autonomously as it will leave the information about ${\hat{q}}_{Sz}^{\left(2\right)}$ contained in ${\hat{q}}_{Mz}^{\left(2\right)}$ unaffected. As such, consider a gate **F**, which is implemented by the unitary $U_{\text{F}}$, and which commutes with ${\hat{q}}_{Sz}^{\left(2\right)}$ and ${\hat{q}}_{Mz}^{\left(2\right)}$ so as to leave these descriptors unaltered. The most general expression of $U_{\text{F}}$ is

5.3 $$U_{\text{F}}({\hat{q}}_{S}^{\left(2\right)},{\hat{q}}_{M}^{\left(2\right)})=\alpha \hat{1}+\beta {\hat{q}}_{Mz}^{\left(2\right)}+\gamma {\hat{q}}_{Sz}^{\left(2\right)}+\delta {\hat{q}}_{Mz}^{\left(2\right)}{\hat{q}}_{Sz}^{\left(2\right)}.$$

The coefficients *α*, *β*, *γ* and *δ* are complex numbers, with the constraint that $U_{\text{F}}$ is unitary, and the effect of the gate **F** on the qubits is

5.4 $$\begin{array}{rl} & {\hat{q}}_{S}^{\left(3\right)}=U_{\text{F}}^{†}({\hat{q}}_{S}^{\left(2\right)},{\hat{q}}_{M}^{\left(2\right)}){\hat{q}}_{S}^{\left(2\right)}U_{\text{F}}({\hat{q}}_{S}^{\left(2\right)},{\hat{q}}_{M}^{\left(2\right)}),\\ & {\hat{q}}_{M}^{\left(3\right)}=U_{\text{F}}^{†}({\hat{q}}_{S}^{\left(2\right)},{\hat{q}}_{M}^{\left(2\right)}){\hat{q}}_{M}^{\left(2\right)}U_{\text{F}}({\hat{q}}_{S}^{\left(2\right)},{\hat{q}}_{M}^{\left(2\right)}). \end{array}\}$$

Because of the form of **F**, both qubits still store the same information about the other qubit as before. For instance, $Q_{S}$ still stores a copy of ${\hat{q}}_{Mz}^{\left(2\right)}$ after the application of the gate, so, by using (5.2), we find that the effect of **F** on that qubit's relative descriptors is

5.5 $$\begin{array}{rl} {\hat{q}}_{S,1}^{\left(3\right)} & \overset{\mathrm{def}}{=}{\hat{q}}_{S}^{\left(3\right)}{\hat{P}}_{1}({\hat{q}}_{Mz}^{\left(2\right)})=U_{{\text{F}}^{\prime }}^{†}({\hat{q}}_{S,1}^{\left(2\right)}){\hat{q}}_{S,1}^{\left(2\right)}U_{{\text{F}}^{\prime }}({\hat{q}}_{S,1}^{\left(2\right)}),\\ {\hat{q}}_{S,-1}^{\left(3\right)} & \overset{\mathrm{def}}{=}{\hat{q}}_{S}^{\left(3\right)}{\hat{P}}_{-1}({\hat{q}}_{Mz}^{\left(2\right)})=U_{{\text{F}}^{\prime \prime }}^{†}({\hat{q}}_{S,1}^{\left(2\right)}){\hat{q}}_{S,-1}^{\left(2\right)}U_{{\text{F}}^{\prime \prime }}({\hat{q}}_{S,1}^{\left(2\right)}), \end{array}\}$$

where the unitaries are defined as $U_{{\text{F}}^{\prime }}({\hat{q}}_{S,1}^{\left(2\right)})\overset{\mathrm{def}}{=}((\alpha +\beta ){\hat{1}}_{S,1}+(\gamma +\delta ){\hat{q}}_{(S,1)z}^{\left(2\right)})$ and $U_{{\text{F}}^{\prime \prime }}({\hat{q}}_{S,-1}^{\left(2\right)})\overset{\mathrm{def}}{=}((\alpha -\beta ){\hat{1}}_{S,-1}+(\gamma -\delta ){\hat{q}}_{(S,-1)z}^{\left(2\right)})$. As can be readily deduced from (5.5), $Q_{S,1}$ and $Q_{S,-1}$ evolve independently between *t* = 2 and *t* = 3, as the instances perform the unconditional single-qubit rotations **F**^′^ and **F**^′′^, respectively. It is similarly possible to express $U_{\text{F}}$ in terms of the relative descriptors of $Q_{M,1}$ and $Q_{M,-1}$ from which it follows, in like manner, that those relative descriptors evolve autonomously under gate **F** too.

Moreover, if the network enacts a single-qubit rotation of $Q_{M}$ or $Q_{S}$ or both (separately), then the relative states evolve independently as well, as such gates do not effect interactions between the qubits and so cannot unentangle them. Thus, the most general gate that ensures the relative states of M evolve autonomously between *t* = 2 and *t* = 3 are products of a gate of the form **F** followed by a single-qubit rotation of either or both qubits. This also makes evident the inherently approximate nature of the relative-state construction: in practice, descriptors will only ever be approximately aligned and the system's dynamics will only approximately preserve this alignment, so a foliation into Everettian universes is never exact.

As we mentioned in §3, the relative-state instances of both $Q_{M}$ and $Q_{S}$ can store locally inaccessible information. Consider, for instance, the case in which the gate $R_{z}(\theta )$ acts on $Q_{M}$ between *t* = 2 and *t* = 3, after which the relative descriptors of $Q_{M,1}$ and $Q_{M,-1}$ depend on the angle *θ*

5.6 $$\begin{array}{rl} {\hat{q}}_{M,1}^{\left(3\right)} & =(\cos\theta {\hat{q}}_{Mx}-\sin\theta {\hat{q}}_{My},\cos\theta {\hat{q}}_{My}+\sin\theta {\hat{q}}_{Mx},{\hat{q}}_{Mz}){\hat{P}}_{1}({\hat{q}}_{Sx}),\\ {\hat{q}}_{M,-1}^{\left(3\right)} & =(\cos\theta {\hat{q}}_{Mx}-\sin\theta {\hat{q}}_{My},-\cos\theta {\hat{q}}_{My}-\sin\theta {\hat{q}}_{Mx},-{\hat{q}}_{Mz}){\hat{P}}_{-1}({\hat{q}}_{Sx}). \end{array}\}$$

The angle *θ* does not appear in any of the expectation values of the relative descriptors since ${\left\langle {\hat{q}}_{M,1}^{\left(3\right)}\right\rangle }_{M,1}=(0,0,1)$ and ${\left\langle {\hat{q}}_{M,-1}^{\left(3\right)}\right\rangle }_{M,-1}=(0,0,-1)$, implying that both $Q_{M,1}$ and $Q_{M,-1}$ must contain information that is not accessible through measurements of either qubit instance alone. However, this information could be retrieved through an interference experiment performed on both branches simultaneously, so the qubit instances store locally inaccessible information. This further exemplifies the fact that a relative state represents a fully quantum system.

## 6. Quasi-classical universes

An Everettian universe is a quantum system. However, when *some* of the relative descriptors that represent an Everettian universe are sharp (with respect to the relative Heisenberg state) and obey quasi-classical equations of motion, then that subset of descriptors represents a *quasi-classical system*. An important example of this is when the relative states of a quantum computational network have sharp *z*-observables that perform a classical computation, as those observables then behave identically to the computational variables of a classical network [20]. It is in this sense that Everettian ‘universes’ can resemble a collection of quasi-classical systems. Even then, as we have said, the spatial extent of these universes can increase only through local interactions, and their relative descriptors do not jointly constitute a description of the full quantum system containing all of them.

To illustrate a foliation into quasi-classical systems, consider, for instance, a quantum computational network C consisting of 2*n* interacting qubits $Q_{1},\;Q_{2},\ldots ,Q_{2n}$. We shall treat C as two subnetworks M and S that consist of the qubits $Q_{1},\;Q_{2},\ldots ,Q_{n}$ and $Q_{n+1},\;Q_{n+2},\ldots ,Q_{2n}$, respectively. We define

6.1 $${\hat{b}}_{M}^{\left(t\right)}\overset{\mathrm{def}}{=}2^{n-1}{\hat{P}}_{-1}({\hat{q}}_{nz}^{\left(t\right)})+\ldots +2{\hat{P}}_{-1}({\hat{q}}_{2z}^{\left(t\right)})+{\hat{P}}_{-1}({\hat{q}}_{1z}^{\left(t\right)}).$$

Since the projectors in (6.1) each have eigenvalues 0 and 1, the eigenvalues of ${\hat{b}}_{M}^{\left(t\right)}$ are binary numbers of length *n*—i.e. elements of $Z_{2^{n}}$, corresponding to memory states of a classical computer. All these memory states, at time *t*, are simultaneously represented by ${\hat{b}}_{M}^{\left(t\right)}$, so when the network performs a classical computation (see below) this descriptor represents the evolution of an *ensemble* of classical computers.

Note that ${\hat{b}}_{M}^{\left(t\right)}$ does not specify the full state of M at time *t* but only the state of the *z*-observables of $\left\{{\hat{q}}_{1}^{\left(t\right)},\ldots ,{\hat{q}}_{n}^{\left(t\right)}\right\}$. And since those remaining descriptors of M could, for instance, store locally inaccessible information not present in ${\hat{b}}_{M}^{\left(t\right)}$, the fully multiversal system M contains more structure than the ensemble of classical computers (or classical ‘universes’) represented by ${\hat{b}}_{M}^{\left(t\right)}$.

We define a similar descriptor for the subnetwork S

6.2 $${\hat{b}}_{S}^{\left(t\right)}\overset{\mathrm{def}}{=}2^{n-1}{\hat{P}}_{-1}({\hat{q}}_{(2n)z}^{\left(t\right)})+\ldots +2{\hat{P}}_{-1}({\hat{q}}_{(n+2)z}^{\left(t\right)})+{\hat{P}}_{-1}({\hat{q}}_{(n+1)z}^{\left(t\right)}).$$

So ${\hat{b}}_{S}^{\left(t\right)}$ has a spectrum of eigenvalues that is equivalent to $Z_{2^{n}}$, just as ${\hat{b}}_{M}^{\left(t\right)}$ does. We shall consider the case in which ${\hat{b}}_{M}^{\left(t\right)}$ can be foliated into an ensemble of classical computers. To that end, let us assume that, at the initial time *t*, subnetwork M is entangled with S. In particular, we assume that the product ${\hat{b}}_{M}^{\left(t\right)}{\hat{b}}_{S}^{\left(t\right)}$ is sharp while separately ${\hat{b}}_{S}^{\left(t\right)}$ and ${\hat{b}}_{M}^{\left(t\right)}$ are non-sharp, as then, following §4, the descriptor ${\hat{b}}_{M}^{\left(t\right)}$ decomposes into relative descriptors, each relative to an eigenvalue *j* of ${\hat{b}}_{S}^{\left(t\right)}$, i.e.

6.3 $${\hat{b}}_{M,j}^{\left(t\right)}\overset{\mathrm{def}}{=}{\hat{b}}_{M}^{\left(t\right)}{\hat{P}}_{j}({\hat{b}}_{S}^{\left(t\right)})\;(j\in Z_{2^{n}}).$$

Here, the projector ${\hat{P}}_{j}({\hat{b}}_{S}^{\left(t\right)})$ projects onto the eigenstate corresponding to the eigenvalue *j* of ${\hat{b}}_{S}^{\left(t\right)}$, and the relative Heisenberg states, associated with each relative descriptor, are normalized products of the projectors $\left\{{\hat{P}}_{j}({\hat{b}}_{S}^{\left(t\right)})\right\}$ and the Heisenberg state |*Ψ*〉.

M is said to perform a reversible classical computation during the (*t* + 1)th computational step if ${\hat{b}}_{M}^{\left(t+1\right)}=f_{t}({\hat{b}}_{M}^{\left(t\right)})$ for some function *f_t_* that maps the spectrum of binary number eigenvalues of ${\hat{b}}_{M}^{\left(t\right)}$ to itself.^9^ And if, between times *t* and *t* + 1, M effects a classical computation without interacting with S, the relative descriptors (6.3) evolve autonomously

6.4 $${\hat{b}}_{M,j}^{\left(t+1\right)}=f_{t}({\hat{b}}_{M,j}^{\left(t\right)})\;(j\in Z_{2^{n}}).$$

As can be ascertained from (6.4), the state of ${\hat{b}}_{M,j}^{\left(t+1\right)}$ depends exclusively on the function *f_t_* and the initial descriptor ${\hat{b}}_{M,j}^{\left(t\right)}$. Thus, if $\left\langle {\hat{P}}_{j}({\hat{b}}_{S}^{\left(t\right)})\right\rangle$ is non-zero, then during the (*t* + 1)th computational step, the information contained in ${\hat{b}}_{M,j}^{\left(t\right)}$ is processed by that relative descriptor alone. Moreover, each relative descriptor ${\hat{b}}_{M,j}^{\left(t\right)}$ has a sharp value with respect to its relative Heisenberg state and has a unique history defined by the function *f_t_*. Which is to say that the relative descriptors ${\hat{b}}_{M,j}^{\left(t\right)}$ describe simultaneously the existing classical systems.

## 7. Conclusion

We have formulated the relative-state construction in the Heisenberg picture. The relative-state foliation is spatially local (can only spread through local interactions), and so are the Everettian ‘universes’, each of which is fully quantum.

## Footnotes

1 Despite the name, an Everettian ‘parallel universe’ only ever has a finite spatial extent, because the Everettian multiplicity arises from, and spreads via, local interactions—as we shall explain.

2 A projector for a degenerate eigenvalue projects onto an *eigenspace*, which is a vector space spanned by the eigenstates that correspond to a given (degenerate) eigenvalue. Measurements of degenerate eigenvalues are described by a straightforward generalization of the model measurement (2.1).

3 Imperfect measurements are described in a similar manner by a so-called positive operator-valued measure, i.e. a set of semi-definite positive operators that sum to unity [4].

4 Everett [1] wrote that ‘*it is not so much the system which is affected by an observation as the observer, who becomes correlated with the system.* [Everett's italics. By ‘correlated’ he means entangled.] Only the observer state has changed, so as to become correlated with the state of the near system and hence naturally with the state of a remote system also’.

5 Networks are also able to model unphysical systems, including, for example, those where the speed of light is exceeded or the principle of locality is violated. Hence, we must envisage mappings between the model and a real system that faithfully implement the latter's laws. For instance, a network model has a preferred rest-frame, yet it can be designed to model systems in a relativistically invariant way by having its input specify both the space–time's metric tensor and the system's state of motion, etc. in the same coordinate system. Thus, when we speak of spatially separated events happening ‘at time *t*’, we are saying that they happen on a space-like hypersurface at coordinate *t* in the modelled quantum system.

6 We call these ‘universes’ to match the terminology used by DeWitt & Graham [15] and Everett (see Byrne [16, ch. 34]). Other authors have used different terms to refer to these Everettian entities, such as ‘worlds’, ‘branches’ or ‘histories’. The different metaphysical connotations of these terms do not concern us here: our construction would apply to any of them. And the Heisenberg picture makes explicit that these ‘universes’, ‘worlds’, ‘branches’ or ‘histories’ are local: as we shall show, immediately after a measurement, the entities that exist in multiple relative states are precisely the system and the measuring instrument, and nothing beyond.

7 Our relative-state construction is local in the sense of Deutsch & Hayden [9], Brassard & Raymond-Robichaud [11] and Einstein [7]. It is not ‘local’ in the sense of, for example, Wallace & Timpson [18] or Kastner [19] because quantum theory itself is not.

8 That two quantum systems can instantiate the same information does not violate Einstein's criterion since the copy of the information in one system can be altered without this affecting the copy in the other system.

9 The function *f_t_* can invariably be implemented by a network consisting exclusively of **ccnot** (Toffoli) gates, as this gate is universal for reversible classical computation [20].

## Data accessibility

This article has no additional data.
